# Initial conceptual development of a pilot questionnaire for patient-perceived clinical inertia in chronic disease management: an exploratory study with a predominantly Peruvian sample

**DOI:** 10.3389/frhs.2026.1839937

**Published:** 2026-07-31

**Authors:** Jorge Isaac Necochea-Chamorro, Monica Elisa Meneses-La-Riva

**Affiliations:** Universidad César Vallejo, Lima, Peru

**Keywords:** chronic diseases, clinical inertia, continuity of care, health systems, patient experience

## Abstract

**Introduction:**

Clinical inertia has traditionally been conceptualized as a physician-centred phenomenon; however, in chronic disease management, it may also be influenced by patient-related factors and structural constraints within healthcare systems, particularly in resource-constrained settings.

**Objective:**

To describe the initial conceptual development of a pilot questionnaire intended to explore patient-perceived clinical inertia in chronic disease management and to report its exploratory pilot-stage performance in terms of content validity, preliminary internal consistency, descriptive item behavior, and preliminary item-grouping patterns.

**Methods:**

A quantitative, cross-sectional, exploratory pilot study was conducted using an online questionnaire disseminated through academic networks and social media in a predominantly Peruvian sample recruited online, with additional responses from other Latin American countries. The questionnaire assessed domains related to medical follow-up, patient knowledge, personal barriers, patient–doctor relationship, and healthcare system barriers. A total of 74 responses were collected, of which 51 complete responses were included. Content validity was examined using Aiken's V, preliminary internal consistency using Cronbach's alpha and McDonald's omega, and exploratory factor analysis was retained only as a preliminary descriptive item-grouping procedure.

**Results:**

The questionnaire showed high content validity based on expert judgment. In this pilot sample, retained dimensions showed preliminary internal consistency estimates within acceptable ranges, whereas the knowledge items showed low reliability and were excluded from the preliminary item-grouping analysis. The preliminary item-grouping analysis showed a tentative four-factor pattern that should be interpreted only as descriptive information for questionnaire refinement. Participants reported perceived delays in treatment adjustment, limited access to medications, prolonged waiting times for specialist care, and administrative barriers affecting continuity of care.

**Conclusions:**

This exploratory pilot study provides initial evidence to inform further refinement of a questionnaire for patient-perceived clinical inertia. The findings should not be interpreted as population-level evidence or as confirmation of structural validity. Future versions should incorporate additional domains related to patient reluctance to treatment change, socioeconomic barriers, polypharmacy, treatment implementation, adverse-effect monitoring, and longitudinal follow-up. Because the sample was small, highly educated, recruited online, and predominantly composed of participants receiving care in Peru, the findings should not be generalized to Latin America or to chronic disease populations more broadly.

## 1 Introduction

Non-communicable chronic diseases (NCDs), such as diabetes mellitus, arterial hypertension, and cardiovascular diseases, constitute one of the main challenges for health systems worldwide due to their high burden of morbidity, mortality, and associated costs (1, 2). Despite pharmacological advances and the widespread availability of evidence-based clinical practice guidelines, a significant proportion of patients fail to achieve recommended therapeutic targets, thereby perpetuating the risk of long-term complications (3, 4).

One of the main determinants of this suboptimal control is the phenomenon known as clinical inertia or therapeutic inertia. Traditionally, this concept has been defined as the failure of healthcare providers to initiate or intensify treatment when explicit therapeutic goals are not achieved (3, 5). Classical literature has predominantly attributed this phenomenon to physician-related factors, including lack of knowledge, misperception of disease control, or insufficient motivation, estimating that these account for a substantial proportion of clinical inertia (4, 5).

However, more recent and systemic perspectives suggest that clinical inertia is not solely attributable to the prescriber, but rather emerges from a complex and multifactorial interaction between physicians, patients, and healthcare systems (4, 6). Studies have identified numerous determinants influencing the quality of care, including organizational, structural, and patient-related factors, reflecting the inherent complexity of chronic disease management (7). Additionally, clinical decision-making often occurs under conditions of uncertainty, where physicians must balance standardized guidelines with individualized patient needs, which may further contribute to clinical inertia (8).

Structural barriers such as healthcare system overload, fragmentation of care, and limited organizational resources further hinder timely therapeutic adjustments (5). This situation is particularly relevant in low- and middle-income countries, such as those in Latin America, where inequities in access to medications and primary care infrastructure pose additional challenges for effective disease management (2, 9).

Although clinical inertia has been widely quantified using objective indicators, such as the time to therapeutic intensification following abnormal clinical or laboratory findings (10), the subjective perspective of patients regarding this phenomenon remains underexplored. In parallel, the growing emphasis on patient-centered care highlights the importance of communication, shared decision-making, and alignment with patient preferences as key components of healthcare quality (11). Evidence also suggests that effective clinician–patient communication is associated with improved adherence and health outcomes (12).

However, when healthcare systems fail to adequately explore patients' perspectives, preferences, and perceived barriers, or when communication remains unidirectional, there is a risk of misinterpreting the causes of poor disease control, thereby perpetuating clinical inertia (13). This reveals an important gap in the literature: while clinical inertia has been extensively studied from a provider-centered perspective, limited attention has been given to how patients perceive and experience the lack of therapeutic adjustments.

Understanding how patients interpret delays or lack of changes in their treatment, as well as the institutional and interpersonal barriers they face, is essential for generating patient-centered evidence on chronic disease management. This study does not attempt to measure clinical inertia in its strict clinical definition based on objective clinical indicators or provider decision-making. Instead, it explores patient-perceived clinical inertia as an exploratory subjective construct reflecting perceived delays, communication barriers, and treatment-adjustment experiences within chronic disease care.

Because patient-perceived clinical inertia is an emerging and complex construct, the present study should be understood as an initial conceptual approximation. The current version focuses on perceived follow-up, communication, personal barriers, doctor–patient interaction, and health-system barriers. However, other relevant domains, including patient reluctance to treatment change, socioeconomic constraints, treatment burden, polypharmacy, treatment implementation, monitoring of adverse effects, and longitudinal follow-up, require further incorporation in subsequent versions of the questionnaire.

The study also does not attempt to validate the instrument definitively. Rather, it reports an initial pilot-stage assessment intended to inform item refinement, dimensional reconsideration, and future validation studies.

In this manuscript, the regional relevance of the study should be understood as an initial context of application in Spanish-speaking Latin American participants, not as evidence representative of Latin America. Because most participants received care in Peru, the findings should be interpreted primarily as exploratory evidence from a predominantly Peruvian sample.

## 2 Methodology

### 2.1 Study design

A quantitative, observational, cross-sectional, exploratory pilot study was conducted to describe the initial development of an instrument for patient-perceived clinical inertia in chronic disease management. The study was designed as a pilot-stage assessment intended to examine content validity, preliminary reliability, descriptive item behavior, and preliminary item-grouping patterns, rather than to establish definitive psychometric validity or produce generalizable estimates. The study also descriptively explored perceived barriers related to treatment adjustment from the patient's perspective. The exploratory nature of the study is justified by the complexity of the phenomenon, its multicausal nature, and the limited empirical evidence focused on the patient's experience in Latin American contexts. Accordingly, this study aims to identify patterns and generate hypotheses rather than establish causal relationships or produce generalizable estimates.

The study was not designed to estimate patient-perceived clinical inertia at a regional level or to provide representative evidence for Latin America.

### 2.2 Instrument development

A structured pilot questionnaire was designed to explore the patient's perception of clinical inertia and the barriers associated with the follow-up, adjustment, and continuity of treatment in chronic diseases. The instrument was developed based on a conceptual review of the literature related to clinical inertia and patient-related dimensions, such as continuity of care, doctor–patient relationship, and health system performance.

A preliminary construct matrix was developed to guide item generation. This matrix included five conceptual domains: perceived medical follow-up, disease knowledge, personal barriers to treatment change, doctor–patient relationship, and health-system barriers. Each domain was linked to a specific conceptual function within the proposed construct. Perceived medical follow-up was intended to capture patient-reported experiences of treatment stagnation or lack of adjustment; disease knowledge captured perceived understanding of disease consequences and treatment; personal barriers captured fears, discomfort, or difficulty expressing dissatisfaction; doctor–patient relationship captured communication, trust, and involvement in decision-making; and health-system barriers captured access, administrative, and continuity-of-care constraints. The preliminary construct matrix is presented in Supplementary Table S1 to improve transparency regarding the conceptual basis of the pilot questionnaire and to allow readers to evaluate the initial coverage of the proposed construct.

The initial construct matrix was intentionally limited to domains considered feasible for a first pilot application. It did not include all possible determinants of perceived clinical inertia. Specifically, the first version did not include separate item domains for patient reluctance to treatment change, socioeconomic barriers, polypharmacy, treatment implementation, short-term monitoring of adverse effects, or longitudinal follow-up. These domains were identified as relevant areas for expansion in future versions of the questionnaire.

Because this was an initial pilot-stage questionnaire, patients were not involved in the item-generation stage through qualitative interviews, focus groups, or cognitive interviewing. We acknowledge that this represents a methodological limitation for a questionnaire intended to assess patient experience. Therefore, cognitive interviewing with patients with chronic diseases will be incorporated as a planned step in the next refinement phase to evaluate item comprehension, relevance, wording, response processes, and domain coverage before formal psychometric validation.

Items were drafted to represent observable patient-reported experiences within each domain. The initial item review considered four criteria: conceptual correspondence with the intended domain, clarity for patients with chronic diseases, relevance to treatment adjustment or continuity of care, and readability in an online self-administered questionnaire. Items considered ambiguous or insufficiently aligned with the intended domain were reformulated before expert review. No items were eliminated at this stage; however, the pilot results and reviewer feedback indicate that the construct matrix requires expansion before formal validation.

The pilot phase focused on clarity, feasibility, and acceptability of the online questionnaire rather than statistical item reduction. Because of the limited sample size, item elimination based on factor loadings was not performed. Instead, the pilot results were used to identify areas requiring conceptual refinement, particularly the knowledge dimension.

The questionnaire was composed of 19 items, organized into six sections: (i) sociodemographic and clinical data, (ii) perception of medical follow-up, (iii) knowledge of the disease, (iv) personal barriers to treatment change, (v) doctor–patient relationship, and (vi) perception of the health system.

The perception-related items were structured using a five-point Likert scale, where 1 corresponds to “Strongly disagree” and 5 to “Strongly agree”.

For each dimension, an index was calculated as the arithmetic mean of the corresponding items. Only complete responses were included; therefore, no imputation for missing data was required. The indices were interpreted descriptively within the exploratory framework of the study. Higher values indicate greater perceived barriers and higher levels of perceived clinical inertia.

### 2.3 Content validity

The instrument's content validity was assessed through expert judgment. The evaluation of the questionnaire was conducted by a panel of four experts, each with professional and research experience in health sciences and chronic disease management. The composition of the panel was as follows: Firstly, a PhD in Nursing specialising in intensive care, with clinical experience in critical patient care and active involvement in academic research. Secondly, a hospital physician with experience in the follow-up and management of patients with chronic diseases. Thirdly, two PhDs in Psychology specialising in research methodology and the measurement of health-related constructs.

Each expert evaluated the items independently according to three criteria: clarity, relevance, and representativeness with respect to the construct under study. The level of agreement among the evaluators was quantified using Aiken's V coefficient, with values above 0.70 considered indicative of adequate content validity.

The results demonstrated a high degree of consensus among the experts on all items, with Aiken's V values ranging from 0.94 to 1.00, thus indicating high content validity based on expert judgment. Detailed results for each item can be found in the Supplementary Material.

The Aiken V was selected as it is a widely utilised metric for quantifying expert agreement in the evaluation of content validity for newly developed instruments (14).

### 2.4 Reliability analysis

Following the content validation process, the questionnaire was administered in a pilot phase to evaluate the clarity and feasibility of the instrument. Following this stage, no modifications were required in the wording, structure, or response scale of the items; therefore, the same instrument was used for the final data collection.

The internal consistency of the scale was evaluated using Cronbach's alpha coefficient and McDonald's omega, based on the 51 valid and complete responses included in the final analytical sample. Cronbach's alpha was used as a traditional indicator of internal consistency (15), whereas McDonald's omega was included as a complementary reliability indicator because it has been recommended as a more general reliability estimate under less restrictive measurement assumptions (16). Cronbach's alpha was calculated for the overall instrument and separately for each questionnaire dimension. McDonald's omega was calculated for the retained dimensions included in the exploratory factor analysis. Given the exploratory nature of the study and the limited sample size, both coefficients were interpreted as preliminary indicators of internal consistency.

In this pilot sample, the overall Cronbach's alpha was *α* = 0.89, suggesting adequate preliminary internal consistency. Reliability coefficients for each retained dimension are presented in Tables 1 and 2, including Cronbach's alpha and McDonald's omega. Most retained dimensions showed preliminary reliability estimates within acceptable ranges; however, these estimates should be interpreted cautiously due to the small sample size and pilot-stage design. The knowledge dimension exhibited low reliability (*α* = 0.25) and was therefore not retained for the exploratory factor analysis. This finding suggests that the items comprising this subscale may represent heterogeneous aspects of patient knowledge and require conceptual refinement in future versions of the instrument.

**Table 1:** Item-level descriptive statistics and internal consistency by dimension.

Dimension/Item	Mean (SD)	% Agreement (4 + 5)
Perception of medical follow-up (*α* = 0.83)		
9. I feel that my treatment has not been modified even though my health has not improved.	3.11 (1.29)	39.2%
10. During consultations, my physician rarely checks whether the treatment remains appropriate.	3.15 (1.25)	45.0%
11. Even after reporting new symptoms, my treatment has not been changed.	2.88 (1.25)	35.2%
12. I have the impression that the physician maintains the same treatment even when new symptoms appear.	3.11 (1.32)	43.1%
Index of Perceived Medical Follow-up = 3.06 (1.28)		
Knowledge of the disease (α = 0.25)		
13. I clearly understand the consequences of not adequately treating my disease.	4.15 (1.20)	82.3%
14. I am able to identify when it might be necessary to change the treatment according to my health evolution.	4.01 (1.12)	72.5%
15. My current treatment has been clearly explained to me.	3.84 (1.13)	70.5%
Disease Awareness Index = 4.0 (1.15)		
Personal barriers (α = 0.82)		
16. I am afraid that a new treatment might cause unwanted side effects.	3.70 (1.17)	64.7%
17. I do not always understand why my physician decides not to change the treatment.	3.45 (1.37)	56.8%
18. I find it difficult to tell my physician that I am not satisfied with the results of the treatment.	3.03 (1.32)	41.1%
19. I avoid discussing treatment changes due to distrust or discomfort.	3.07 (1.23)	39.2%
Personal Barriers Index = 3.31 (1.27)		
Doctor-patient relationship (α = 0.87)		
20. I feel that my physician listens to my opinions during the consultation.	3.76 (1.03)	70.5%
21. I can speak openly with my physician about how I feel about the treatment.	3.86 (1.09)	68.6%
22. I trust that the healthcare professional suggests what is best for my condition.	3.80 (1.03)	70.5%
23. During medical care I feel involved in the decisions about my treatment.	3.56 (1.15)	62.7%
Doctor-Patient Relationship Index = 3.74 (1.07)		
Health system barriers (α = 0.74)		
24. The health system delays medical care, preventing my treatment from being monitored on time.	3.78 (1.22)	70.5%
25. I have had difficulties accessing medications when the physician has recommended a change.	3.60 (1.29)	66.6%
26. Delays in scheduling specialist appointments negatively affect the follow-up of my disease.	4.25 (0.86)	88.2%
27. Administrative procedures make it difficult for me to receive the appropriate treatment on time.	4.17 (1.10)	82.3%
Index of Health System Barriers = 3.95 (1.11)		

The knowledge items are reported descriptively only and were not retained as a reliable subscale because of very low internal consistency.

**Table 2:** Dimension-level descriptive and reliability statistics.

Dimension	Items included	Mean (SD)	Cronbach's α	McDonald's *ω*	Status in preliminary item-grouping analysis
Perceived medical follow-up	9–12	3.06 (1.28)	0.83	0.76	Included
Personal barriers	16–19	3.31 (1.27)	0.82	0.75	Included
Doctor-patient relationship	20–23	3.74 (1.07)	0.87	0.85	Included
Health system barriers	24–27	3.95 (1.11)	0.74	0.72	Included

In Table 2, Dimension scores were calculated as the arithmetic mean of the corresponding items. Higher scores indicate greater perceived barriers or higher perceived lack of treatment adjustment.

The knowledge items were retained only for descriptive reporting because they were part of the administered questionnaire. However, due to their very low internal consistency, they were not retained as a reliable subscale and should not be interpreted as a coherent dimension of the pilot questionnaire.

### 2.5 Data collection and unit of analysis

Data were collected between November 2025 and January 2026 using an online questionnaire disseminated via academic contacts and social media platforms, including Facebook, LinkedIn, and WhatsApp, in Peru and other Latin American countries. The sample was obtained using non-probabilistic convenience sampling, consistent with the exploratory nature of the study. Eligible participants were adults aged 18 years or older who reported receiving medical care for at least one chronic disease. Responses with incomplete questionnaires were excluded from the final analysis.

Participants who received care in multiple health institutions were instructed to complete the questionnaire considering one institution at a time, anchoring their responses to a specific healthcare experience. This approach was intentionally adopted to capture the patient's experience of care within a particular institutional context, rather than to classify participants according to the type of health system (e.g., public or private). Consequently, respondents who received care in multiple institutions could report more than one experience, with each experience associated with a different healthcare setting. Each response was treated as an independent patient–institution care experience.

Due to the anonymous nature of the responses, it was not possible to ascertain whether some participants completed the questionnaire on more than one occasion. Therefore, the unit of analysis was defined as the patient–institution care experience rather than the individual participant. This may introduce potential clustering effects or over-representation of individuals who interact more frequently with healthcare services.

Because no participant identifiers were collected, it was not possible to determine how many individuals contributed more than one patient–institution experience. Therefore, no descriptive count of repeated institutional experiences could be calculated. This issue is acknowledged as a limitation of the pilot design because repeated responses from the same individual could introduce clustering effects or over-representation of participants with multiple healthcare experiences.

### 2.6 Statistical analysis

Descriptive statistics were calculated for all items and dimensions, including means, standard deviations, and percentage agreement (responses 4 and 5 on the Likert scale). Agreement was defined as the proportion of participants selecting 4 (Agree) or 5 (Strongly agree), as a descriptive summary of positive endorsement for each Likert-scale statement. Dimension scores were calculated as the arithmetic mean of the corresponding items. Only complete responses were included; therefore, no imputation for missing data was required.

Internal consistency was assessed using Cronbach's alpha for the overall instrument and each dimension. McDonald's omega was also calculated as a complementary reliability indicator for the retained dimensions included in the exploratory factor analysis. The knowledge dimension was excluded from the exploratory factor analysis due to low internal consistency (*α* = 0.25), suggesting that its items did not represent a coherent unidimensional construct in this sample.

An exploratory factor analysis (EFA) was conducted only as a preliminary item-grouping analysis to describe tentative item behavior in the pilot sample 17. Because the sample-to-item ratio was low, the factor analysis was not used as evidence of dimensionality, structural validity, or construct validity 17, 18. It was retained only as a transparent descriptive item-grouping exercise to inform future refinement. Sampling adequacy was assessed using the Kaiser–Meyer–Olkin measure, and Bartlett's test of sphericity was used to evaluate factorability. Factors were extracted using principal axis factoring because the analysis aimed to explore latent dimensions underlying the observed questionnaire items. Varimax rotation was used to obtain a simpler and more interpretable factor structure. Factor retention was guided by eigenvalues greater than 1, factor interpretability, and visual inspection of the scree plot. Factor loadings, cross-loadings, communalities, and explained variance were examined to improve transparency of the factor solution. Accordingly, the EFA was not used to make validation claims, but only to identify tentative item-grouping patterns that may guide future refinement and formal validation.

### 2.7 Ethical considerations

Participation in the study was voluntary and anonymous. No personal data were collected that could identify the participants or the health institutions where they received care.

Prior to the completion of the questionnaire, the participants were informed about the objectives of the study and provided their informed consent to participate. The responses were collected anonymously and used exclusively for research purposes.

The study protocol was reviewed and approved by the institutional ethics committee under Report No. 00469–2025/CEI-EIS.

## 3 Results

### 3.1 Participant characteristics

Table 3 presents the sociodemographic and clinical characteristics of the participants. The sample had a mean age of 53.2 years (SD = 15.8), with a slight majority of male participants (54.9%). Most participants had higher education (university or postgraduate degree: 64.6%) and received medical care in Peru (84.3%), primarily in the country's capital (50.9%). The mean time since diagnosis of chronic disease was 9.25 (SD = 8.46), with median of 7.0 years (IQR = 3.0–12.0). Regarding the main chronic condition, 33.3% of participants reported diseases other than the classic cardiometabolic conditions, while hypertension (23.5%), diabetes type 2 (17.6%), obesity (13.7%), and dyslipidemia (5.8%) were also frequently mentioned.

**Table 3:** Sociodemographic and clinical characteristics of participants (*N* = 51).

Characteristic	Category	n (%)
Age (years)	Mean (SD)	53.2 (15.8)
	Median (IQR)	52.0 (46.5–64.5)
	Range	20–92
Sex	Female	23 (45.09%)
	Male	28 (54.91%)
Education level	Secondary	6 (11.7%)
	Technical	12 (23.5%)
	University	16 (31.3%)
	Postgraduate	17 (33.3%)
Country of care	Peru	43 (84.3%)
	Mexico	6 (11.7%)
	Other	2 (3.9%)
Area of care	Country's capital	26 (50.9%)
	Another major city	17 (33.3%)
	Intermediate city	8 (15.6%)
Time since diagnosis	Mean (SD) years	9.25 (8.46)
	Median (IQR) years	7.0 (3.0–12.0)
Main chronic disease	Hypertension	12 (23.5%)
	Diabetes type 2	9 (17.6%)
	Obesity	7 (13.7%)
	Dyslipidemia	3 (5.8%)
	Arrhythmia	2 (3.9%)
	Chronic kidney disease and heart failure	1 (1.96%)
	Others	17 (33.3%)

SD, Standard Deviation; IQR, Interquartile Range.

### 3.2 Item-level and dimension-level results

Table 1 presents the descriptive statistics and internal consistency for each item and dimension. The highest levels of agreement were observed in the health system barriers dimension, particularly regarding delays in specialist appointments (88.2% agreement) and administrative procedures (82.3% agreement). The perception of medical follow-up dimension showed moderate agreement (35.2%–45.0%), indicating that a substantial minority of patients perceived a lack of timely treatment adjustments. Regarding personal barriers, fear of side effects was the most common (64.7% agreement). The doctor–patient relationship dimension showed moderate-to-high endorsement, with agreement ranging from 62.7% to 70.5% across items. Although high levels of agreement were observed in items related to disease knowledge, this dimension showed low internal consistency (*α* = 0.25) and was therefore excluded from subsequent factor analysis. Preliminary reliability estimates for the retained dimensions ranged from *α* = 0.74 to 0.87 and *ω* = 0.72 to 0.85; however, these estimates should be interpreted cautiously due to the pilot-stage design and small sample size.

### 3.3 Preliminary item-grouping analysis

The Kaiser–Meyer–Olkin measure indicated adequate sampling adequacy (KMO = 0.71), and Bartlett's test of sphericity was significant (*p* < 0.001), suggesting that the correlation matrix was appropriate for this preliminary exploratory analysis.

The preliminary item-grouping analysis showed an interpretable but unstable four-factor pattern with eigenvalues greater than 1. Given the low sample-to-item ratio, this pattern should not be interpreted as evidence supporting the questionnaire's dimensional structure. Based on the rotated sums of squared loadings, this tentative four-factor pattern explained approximately 47.9% of the variance, with factor-specific explained variance of approximately 14.5% for the doctor–patient relationship factor, 11.3% for the medical follow-up factor, 10.8% for the personal barriers factor, and 11.3% for the health system barriers factor.

A summary of the factor interpretation is provided in Table 4. The rotated factor matrix is presented in Table 5 and suggested an interpretable item-grouping pattern in this pilot sample. Factor 1 corresponded to the doctor-patient relationship, Factor 2 to perceived lack of treatment adjustment (medical follow-up), Factor 3 to personal barriers, and Factor 4 to health system barriers.

**Table 4:** Factor interpretation.

Factor	Dimension name	Description
Factor 1	Doctor–patient relationship	Communication and trust in healthcare interaction
Factor 2	Medical follow-up	Lack of treatment adjustment despite clinical need
Factor 3	Personal barriers	Psychological and communication barriers
Factor 4	Health system barriers	Structural and administrative barriers

**Table 5:** Preliminary item-grouping analysis based on exploratory factor analysis.

Item	Factor 1	Factor 2	Factor 3	Factor 4	Communality (h2)
9. I feel that my treatment has not been modified even though my health has not improved		0.39		0.45	0.35
10. During consultations, my physician rarely checks whether the treatment remains appropriate.		0.61			0.37
11. Even after reporting new symptoms, my treatment has not been changed.		0.86			0.74
12. I have the impression that the physician maintains the same treatment even when new symptoms appear.		0.74			0.55
16. I am afraid that a new treatment might cause unwanted side effects.			0.59		0.35
17. I do not always understand why my physician decides not to change the treatment.			0.56		0.31
18. I find it difficult to tell my physician that I am not satisfied with the results of the treatment.			0.79		0.62
19. I avoid discussing treatment changes due to distrust or discomfort.			0.67		0.45
20. I feel that my physician listens to my opinions during the consultation.	0.80				0.64
21. I can speak openly with my physician about how I feel about the treatment.	0.78				0.61
22. I trust that the healthcare professional suggests what is best for my condition.	0.69				0.48
23. During medical care I feel involved in the decisions about my treatment.	0.77				0.59
24. The health system delays medical care, preventing my treatment from being monitored on time.				0.67	0.45
25. I have had difficulties accessing medications when the physician has recommended a change.				0.50	0.25
26. Delays in scheduling specialist appointments negatively affect the follow-up of my disease.				0.70	0.49
27. Administrative procedures make it difficult for me to receive the appropriate treatment on time.				0.64	0.41

KMO = 0.71; Bartlett's test *p* < 0.001; principal axis factoring with varimax rotation. Factor loadings below 0.40 are not shown. Communalities are reported as h^2^.

Most items showed loadings above 0.40 on their respective factors. Communalities ranged from 0.25 to 0.74, indicating variable but generally interpretable item representation within the extracted factors. Minor cross-loadings were observed in a small number of items and were interpreted cautiously due to the exploratory design and limited sample size.

The scree plot showed that the first four factors had eigenvalues greater than 1, followed by a decline below this threshold from the fifth factor onward. This pattern, together with the interpretability of the rotated solution, was consistent with a tentative four-factor pattern; however, this result should be interpreted cautiously and requires replication in larger samples.

## 4 Discussion

### 4.1 General interpretation

The present study explored patient-perceived clinical inertia in the management of chronic diseases, addressing dimensions related to medical follow-up, patient-related factors, the doctor–patient relationship, and institutional barriers within the healthcare system.

Overall, although classical definitions of clinical inertia—such as those revised by Reach et al.—have attributed up to 50% of the burden of inertia to provider factors and defined it primarily as the failure to initiate or intensify treatment when clinically indicated (5), the findings from this pilot sample are consistent with the possibility that patient-perceived treatment delays may involve patient-, provider-, and system-level factors. However, because the study was exploratory and descriptive, these results should be interpreted as signals for further investigation rather than evidence of causal or population-level patterns (19).

In this pilot sample, participants reported perceptions of delayed or insufficient treatment adjustment, particularly when symptoms persisted. While classical literature—following definitions such as those proposed by Phillips et al.—has conceptualized clinical inertia mainly as a failure by healthcare professionals to intensify therapy (10), these descriptive findings may indicate that the traditional provider-centered perspective does not fully capture how some patients experience treatment-adjustment processes within healthcare systems.

Importantly, the findings of this study should be interpreted within the framework of perceived clinical inertia, rather than clinical inertia in its strict clinical definition. While traditional definitions focus on the failure of healthcare providers to initiate or intensify treatment when clinically indicated, the present study captures the patient's subjective interpretation of delays or lack of adjustment in treatment. These perceptions may be influenced not only by clinical decision-making, but also by systemic barriers, communication dynamics, and relational factors within the healthcare system.

### 4.2 Role of the system

Participants frequently identified institutional barriers as important elements shaping their perceptions of delays or limitations in treatment adjustment. Patients reported delays in medical care, administrative complexity, and difficulties accessing medications as frequently reported barriers to adequate treatment follow-up.

These findings align with contemporary literature proposing that clinical inertia should not be understood solely as a failure in medical prescription but also as a consequence of structural problems within healthcare systems, which may shape how patients perceive delays in treatment adjustment. Authors such as Yánez-Cadena et al. argue that a systemic vision that transcends simple clinical intervention is essential to improve the control of chronic diseases (1).

Coinciding with recent reviews, the perceptions reported in this exploratory study suggest that treatment-adjustment experiences may also be influenced by structural and organizational barriers. Usherwood identifies high patient volume and limited consultation time as important factors that hinder therapeutic intensification (20), while Reach et al. point out that competing demands and lack of organizational resources interfere with physicians' ability to adhere to clinical guidelines (5).

In addition, the perception of lack of clinical improvement reported by patients may be exacerbated by fragmented and discontinuous care, typical of current models of chronic disease management, as described by Yánez-Cadena et al. (1). In the context of Latin America, these findings resonate with evidence reported by Brettler et al. in the HEARTS initiative, which highlights persistent barriers in medication availability and access to care in low- and middle-income countries (9). Likewise, the World Heart Federation roadmap emphasizes that inequities in access and insufficient primary care infrastructure constitute key obstacles preventing effective implementation of clinical guidelines (2).

Therefore, exploring patient-perceived clinical inertia requires considering broader systemic factors that may affect continuity and quality of chronic disease care.

### 4.3 The patient's role

Regarding patient-related factors, a relevant distinction emerged between perceived disease knowledge and patient-level barriers to treatment change. Although most participants reported adequate perceived knowledge about their disease and treatment, several patient-level barriers were also identified, including fear of treatment side effects, difficulty understanding medical decisions, and challenges in expressing dissatisfaction with therapeutic outcomes. This finding does not necessarily indicate a contradiction; rather, it suggests that perceived disease knowledge, treatment understanding, communication confidence, and readiness to discuss treatment modification may represent distinct domains of patient experience.

Previous research on health literacy in chronic disease populations has shown that patient knowledge may be fragmented across different domains, including disease understanding, treatment awareness, and self-management skills (21).

These descriptive findings are consistent with previous studies suggesting that patient participation and communication skills are critical elements in avoiding situations of therapeutic inertia, particularly in chronic diseases that require continuous treatment adjustments. In line with Rodriguez and Pantalone, ineffective communication practices and unresolved patient fears can act as powerful barriers to treatment intensification (10).

The perceived difficulty in expressing dissatisfaction with treatment decisions may also reflect a limited participation of patients in clinical decision-making. Perpetua et al. describe shared decision-making as a “two-way exchange between experts,” where the patient contributes knowledge about personal values, fears, and preferences (13). When clinicians fail to actively explore these preferences, patient silence may be misinterpreted as acceptance, which may inadvertently perpetuate therapeutic inertia.

### 4.4 Doctor-patient relationship

The heterogeneity observed in the doctor–patient relationship suggests that the quality of clinical interaction may shape patients' perceptions of care continuity. While some participants reported positive experiences characterized by listening and trust, others described communication difficulties that limited their participation in treatment decisions.

These findings reinforce the evidence presented by Chew et al., who identify lack of professional rapport and trust as root causes of inertia at the patient level (4). Poor communication practices and limited patient engagement may therefore contribute to delays in treatment intensification.

Encouraging shared decision-making (SDM), understood as a bidirectional exchange in which patient values and preferences are recognized, may represent an important strategy to reduce perceived barriers to treatment modification (13).

It is also important to note that this study focused primarily on the role of the physician as the main clinical caregiver, as reflected in the questionnaire items (e.g., “my doctor listens,” “I trust the healthcare professional”). However, in many chronic disease care settings—particularly in primary care—other professionals such as nurses, health educators, and nurse practitioners play a fundamental role in patient education, follow-up, and continuity of care (1, 4, 9, 10, 22–24). Future research should therefore explore the influence of the entire multidisciplinary team on patient perceptions of treatment inertia and access barriers.

Given the relevance of medication access and treatment adjustment, pharmaceutical care may also play an important role in reducing perceived barriers to treatment optimization. Pharmacists can contribute to medication counseling, adherence support, detection of access problems, and communication with the healthcare team, particularly in chronic disease management. Future versions of the instrument should therefore consider incorporating items that capture the perceived role of pharmacists, nurses, health educators, and other members of interprofessional care teams.

### 4.5 Implications for public health

Overall, the findings support the relevance of considering a systemic perspective when exploring patient-perceived clinical inertia beyond the traditional physician-centered approach. Consistent with the multifactorial model proposed by O'Connor et al. and Chew et al., the results suggest that factors related to patients, healthcare professionals, and institutional structures may interact in shaping perceptions of treatment inertia (4).

From a public health perspective, this approach may help generate hypotheses about how patients perceive barriers in chronic disease management. However, its potential utility as an indicator of perceived barriers requires further conceptual refinement, patient-informed item development, and psychometric validation.

The present pilot study may contribute to the emerging literature on patient-perceived clinical inertia in Latin American healthcare systems by highlighting the importance of incorporating patient perspectives in the evaluation of healthcare delivery processes.

This study proposes an initial pilot-stage operationalization of patient-perceived clinical inertia. This operationalization should be considered provisional and intended for further refinement rather than as a finalized measurement model. This conceptualization integrates not only clinical decision-making processes, but also the influence of communication dynamics, patient experiences, and structural constraints within healthcare systems.

### 4.6 Domains requiring expansion in future versions of the questionnaire

Although the present questionnaire included items related to fear of side effects, difficulty understanding medical decisions, and difficulty expressing dissatisfaction, it did not fully capture active patient reluctance to treatment change, including reluctance to modify diet, lifestyle, or complex medication regimens. This is an important conceptual limitation because patient-perceived clinical inertia may arise not only from provider or system delays, but also from patient preferences, concerns, treatment burden, and reluctance to adopt new therapeutic recommendations.

Future versions should also include items assessing whether treatment changes were actually implemented, whether patients received short-term follow-up after treatment modification, whether adverse effects were monitored, and whether socioeconomic barriers or polypharmacy influenced adherence or treatment decisions.

In addition, future refinement should include cognitive interviewing with patients with chronic diseases to examine whether the items are understood as intended, whether the response options are appropriate, and whether the proposed domains adequately reflect patients’ lived experiences of treatment adjustment, follow-up, communication, and access barriers.

### 4.7 Limitations

The findings should be interpreted considering the exploratory nature of the study and the relatively small sample size. Additionally, the use of convenience sampling through online platforms may limit the representativeness of the sample and introduce potential selection biases.

The sample also had a high educational level and was composed mainly of respondents who received care in Peru. In addition, online recruitment through academic networks and social media may have favored the participation of individuals with greater digital access, health literacy, or engagement with healthcare issues. Therefore, the findings should not be interpreted as representative of the broader chronic disease population in Latin America, but rather as preliminary and hypothesis-generating.

The preliminary reliability and item-grouping findings should also be interpreted cautiously. Although the preliminary item-grouping analysis showed an interpretable pattern, these results do not constitute evidence of dimensionality or structural validity. McDonald's omega complemented Cronbach's alpha as a preliminary reliability estimate for the retained item groups; however, these coefficients should also be interpreted cautiously due to the small sample size and possible instability of inter-item correlations. Future studies using larger and more diverse samples are required to evaluate the dimensional structure using adequately powered psychometric designs and to examine additional psychometric properties.

Second, the data are based on self-reported information. Although this approach allows capturing the patient's perspective, self-reported responses may be influenced by recall or perception biases.

Third, the cross-sectional design of the study prevents establishing causal relationships between the variables analyzed.

Another limitation concerns the absence of objective clinical information, such as laboratory or physiological indicators, which could allow direct comparison between patient perceptions and clinical outcomes. This limitation is particularly relevant given that the study focuses on perceived clinical inertia, as patient-reported experiences may not necessarily reflect objectively measurable clinical decision-making processes.

After excluding the knowledge dimension, the EFA was conducted on 16 items with 51 complete responses, corresponding to approximately 3.2 participants per item. Although sample-size requirements in factor analysis depend on several features of the data, including communalities and factor overdetermination, this ratio is below commonly recommended thresholds for stable factor solutions. Therefore, considering methodological recommendations for EFA and sample size in factor analysis, the tentative item-grouping pattern was interpreted strictly as preliminary and descriptive (17, 18).

Because a substantially larger and more diverse sample was not available for the present revision, the current manuscript should be interpreted strictly as an initial exploratory pilot study. A full validation study remains necessary before the instrument can be recommended for broader research or clinical use. Such a study should include a larger and more heterogeneous sample, reassessment of the tentative factor pattern, reconceptualization of the knowledge dimension, and evaluation of additional psychometric properties using a more robust validation model.

Finally, this study does not measure clinical inertia in its strict clinical sense, which traditionally refers to the failure of healthcare providers to initiate or intensify treatment when clinically indicated. Instead, it focuses on patient-perceived clinical inertia, defined as the patient's perception that treatment adjustments or follow-up are delayed or constrained by systemic, relational, or communication barriers. Therefore, these perceptions should be interpreted within the subjective and exploratory nature of the construct.

The knowledge dimension showed low internal consistency (*α* = 0.25), suggesting that it may represent a multidimensional construct.

This dimension should therefore not be interpreted as a reliable reflective subscale in its current form. Its low internal consistency suggests that the items may capture heterogeneous or potentially formative aspects of disease awareness, treatment understanding, and recognition of treatment-change needs. Future versions of the instrument should revise or reconceptualize this dimension before further validation.

Because responses were anonymous, it was not possible to determine whether some participants contributed more than one patient–institution experience. This may have introduced clustering or over-representation of individuals with multiple healthcare experiences. No sensitivity analysis could be performed because participant identifiers were not collected. Consequently, the results should be interpreted at the level of reported care experiences rather than as independent individual-level patient observations.

If some individuals contributed more than one patient–institution experience, the assumption of independent observations may have been violated. This could have affected the estimation of internal consistency coefficients and the correlation matrix used for the preliminary item-grouping analysis, potentially inflating or distorting reliability estimates and factor loadings. Because participant identifiers were not collected, clustering could not be assessed or adjusted for. Future studies should collect non-identifying participant codes to distinguish individuals from care experiences and allow sensitivity analyses.

A further limitation concerns the incomplete coverage of the construct. The first version of the questionnaire did not include specific domains assessing patient reluctance to treatment change, socioeconomic constraints, polypharmacy, treatment burden, treatment implementation, short-term adverse-effect monitoring, or longitudinal follow-up. Therefore, the current questionnaire should be interpreted as an initial item pool rather than as a comprehensive measure of patient-perceived clinical inertia.

A further methodological limitation is that patients were not involved in the initial item-generation stage through qualitative interviews, focus groups, or cognitive interviewing. For a questionnaire intended to assess patient experience, this limits the qualitative grounding of the item pool and may have affected the comprehensiveness, wording, and patient relevance of the initial domains. Future refinement should therefore include patient-informed qualitative procedures before formal validation.

Although the EFA was retained only as a transparent preliminary item-grouping procedure, the small sample size limits the stability of the observed pattern. Future validation studies should involve larger samples and statistical review during the design and analysis stages.

Consequently, the present findings should be interpreted as evidence for refining the construct and item pool, not as evidence supporting the readiness of the questionnaire for applied clinical or epidemiological use.

This limitation is structural rather than merely procedural, because patient-perceived clinical inertia is intended to capture subjective patient experience. Therefore, the current item pool should be considered expert-informed and literature-informed, but not yet patient-informed.

## 5 Conclusions

This exploratory pilot study describes an expert-informed and literature-informed initial item pool for a pilot questionnaire for assessing patient-perceived clinical inertia in chronic disease management. The pilot findings provided preliminary information on content validity, internal consistency, item behavior, and a tentative factor pattern; however, they do not constitute definitive psychometric validation or confirmation of structural validity.

The questionnaire should not yet be considered patient-informed or structurally validated.

In this pilot sample, participants reported perceived barriers related to treatment adjustment, including access to care and medications, administrative procedures, continuity of care, and communication with healthcare professionals. These descriptive findings should be interpreted as preliminary signals to guide further instrument refinement rather than as generalizable evidence about patient-perceived clinical inertia in Latin America.

Further studies with larger, more diverse, and clinically characterized samples are required to refine the instrument, reassess the knowledge items, evaluate the dimensional structure using adequately powered psychometric designs, and examine additional psychometric properties.

Therefore, the questionnaire should be considered a pilot-stage item pool requiring substantial conceptual refinement and psychometric validation before use as a validated measure of patient-perceived clinical inertia.

Before formal validation, the questionnaire requires conceptual expansion to include patient reluctance to treatment change, socioeconomic barriers, polypharmacy, treatment burden, implementation of treatment changes, adverse-effect monitoring, and longitudinal follow-up.

### 5.1 Implications and future research

Subsequent studies should incorporate patient-informed qualitative procedures, including cognitive interviewing, followed by larger and probabilistic samples, longitudinal designs, and objective clinical information that allows comparison between patient perceptions and clinical indicators.

Future validation studies should also consider confirmatory factor analysis or other robust psychometric models, assessment of measurement invariance across healthcare contexts, and comparison between patient-reported perceptions and objective clinical indicators of treatment intensification.

## Data Availability

The raw data supporting the conclusions of this article will be made available by the authors, without undue reservation.
