# Supplementary material for: Initial conceptual development of a pilot questionnaire for patient-perceived clinical inertia in chronic disease management: an exploratory study with a predominantly Peruvian sample
Source: Front Health Serv. 2026 Jul 31;6:1839937. doi: 10.3389/frhs.2026.1839937 (PMC13473296; doi:10.3389/frhs.2026.1839937)
Supplement: Supplementary file 1 [file Table1.docx]

Supplementary Material

# 1 Supplementary Table S1. Preliminary construct matrix used for item generation

| Domain | Conceptual function | Items | Rationale |
| --- | --- | --- | --- |
| Perceived medical follow-up | Captures perceived treatment stagnation or lack of adjustment | 9–12 | Represents patient-reported experiences of insufficient treatment review, lack of modification despite persistent symptoms, or perceived continuation of the same treatment despite lack of improvement. |
| Disease knowledge | Captures perceived understanding of disease consequences and treatment | 13–15 | Represents patients’ self-perceived understanding of chronic disease, treatment, and possible need for treatment change; later identified as heterogeneous in the pilot analysis. |
| Personal barriers to treatment change | Captures fears, discomfort, or difficulty expressing dissatisfaction | 16–19 | Represents patient-level barriers that may limit discussion of treatment adjustment, intensification, or dissatisfaction with therapeutic outcomes. |
| Doctor–patient relationship | Captures communication, trust, and involvement in decision-making | 20–23 | Represents the relational and communicative context in which treatment decisions are discussed between patients and healthcare professionals. |
| Health-system barriers | Captures access, administrative, and continuity-of-care constraints | 24–27 | Represents structural barriers that may delay follow-up, medication access, specialist care, or timely treatment adjustment. |

# 2 Supplementary File 1. Questionnaire used to assess patient-perceived clinical inertia in chronic disease management

Note: The questionnaire is presented in Spanish because it was administered to Spanish-speaking participants in Peru and other Latin American countries.

Cuestionario sobre experiencias de atención médica en enfermedades crónicas

Estimado(a) participante:

Este cuestionario está dirigido exclusivamente a personas con enfermedades crónicas. Si no se encuentra dentro de este grupo, le solicitamos no completar el formulario.

El objetivo de este cuestionario es conocer las experiencias, percepciones y opiniones de los pacientes respecto a su atención médica, el manejo de su enfermedad crónica y el acceso a los servicios de salud.

Su participación es voluntaria y anónima. No existen respuestas correctas o incorrectas; lo importante es su experiencia personal.

Si recibe atención en más de una institución de salud, le solicitamos responder el cuestionario considerando una institución a la vez, preferentemente aquella en la que se atiende con mayor frecuencia o donde haya tenido una experiencia más significativa en los últimos meses. En caso de desearlo, puede completar nuevamente el cuestionario considerando otra institución en la que también reciba atención.

Por favor, responda cada ítem de acuerdo con su experiencia personal, utilizando una escala de 1 (totalmente en desacuerdo) a 5 (totalmente de acuerdo). El tiempo estimado de respuesta es de 8 a 10 minutos.

Al completar este cuestionario, usted autoriza el uso anónimo de sus respuestas con fines de investigación académica.

Sección I: Datos sociodemográficos y clínicos

1. Enfermedad crónica principal (Seleccione una opción)

☐ Diabetes mellitus tipo 2

☐ Hipertensión arterial

☐ Dislipidemias (colesterol alto, dislipidemia mixta)

☐ Asma / enfermedad pulmonar obstructiva crónica (EPOC)

☐ Obesidad

☐ Enfermedad renal crónica e insuficiencia cardiaca

☐ Otro

☐ Ninguna

2. Si seleccionó “Otro” en la pregunta anterior, indique su enfermedad crónica principal

3. Edad (en años): _____

4. Sexo (Seleccione una opción)

☐ Masculino

☐ Femenino

☐ Prefiero no decirlo

5. Nivel educativo alcanzado (Seleccione una opción)

☐ Sin estudios

☐ Primaria

☐ Secundaria

☐ Técnica

☐ Universitaria

☐ Posgrado

6. Tiempo desde el diagnóstico de su enfermedad crónica (en años cumplidos): ____

7. País donde recibe habitualmente atención médica (Seleccione una opción)

☐ Perú

☐ México

☐ Otro

8. Tipo de zona donde recibe atención médica (Seleccione una opción)

☐ Capital del país

☐ Otra ciudad principal / metropolitana

☐ Ciudad intermedia

☐ Zona rural

Instrucciones para las siguientes secciones:

Lea cada afirmación y marque la opción que mejor representa su opinión:

1 = Totalmente en desacuerdo

2 = En desacuerdo

3 = Ni de acuerdo ni en desacuerdo

4 = De acuerdo

5 = Totalmente de acuerdo

Sección II: Percepción del seguimiento médico

9. Siento que mi tratamiento médico no ha sido ajustado, aunque mi salud no ha mejorado (Seleccione una opción)

☐ Totalmente de acuerdo

☐ De acuerdo

☐ Ni de acuerdo ni en desacuerdo

☐ En desacuerdo

☐ Totalmente en desacuerdo

10. Durante las consultas, mi médico rara vez revisa si el tratamiento sigue siendo adecuado (Seleccione una opción)

# ☐ Totalmente de acuerdo

# ☐ De acuerdo

# ☐ Ni de acuerdo ni en desacuerdo

# ☐ En desacuerdo

# ☐ Totalmente en desacuerdo

11. Aunque he informado de nuevos síntomas, mi tratamiento no ha cambiado (Seleccione una opción)

☐ Totalmente de acuerdo

☐ De acuerdo

☐ Ni de acuerdo ni en desacuerdo

☐ En desacuerdo

☐ Totalmente en desacuerdo

12. Tengo la impresión de que el médico prefiere mantener el mismo tratamiento por mucho tiempo (Seleccione una opción)

☐ Totalmente de acuerdo

☐ De acuerdo

☐ Ni de acuerdo ni en desacuerdo

☐ En desacuerdo

☐ Totalmente en desacuerdo

Sección III: Conocimiento de su enfermedad

13. Entiendo bien cuáles son las consecuencias de no tratar mi enfermedad de manera adecuada (Seleccione una opción)

☐ Totalmente de acuerdo

☐ De acuerdo

☐ Ni de acuerdo ni en desacuerdo

☐ En desacuerdo

☐ Totalmente en desacuerdo

14. Sé cuando podría ser necesario cambiar el tratamiento según mi evolución (Seleccione una opción)

☐ Totalmente de acuerdo

☐ De acuerdo

☐ Ni de acuerdo ni en desacuerdo

☐ En desacuerdo

☐ Totalmente en desacuerdo

15. Me han explicado claramente cómo actúa mi tratamiento actual (Seleccione una opción)

☐ Totalmente de acuerdo

☐ De acuerdo

☐ Ni de acuerdo ni en desacuerdo

☐ En desacuerdo

☐ Totalmente en desacuerdo

Sección IV: Barreras personales para el cambio de tratamiento

16. Siento temor de que un nuevo tratamiento me cause efectos secundarios indeseados (Seleccione una opción)

☐ Totalmente de acuerdo

☐ De acuerdo

☐ Ni de acuerdo ni en desacuerdo

☐ En desacuerdo

☐ Totalmente en desacuerdo

17. No siempre entiendo por qué mi médico decide no cambiar el tratamiento (Seleccione una opción)

☐ Totalmente de acuerdo

☐ De acuerdo

☐ Ni de acuerdo ni en desacuerdo

☐ En desacuerdo

☐ Totalmente en desacuerdo

18. Me cuesta expresar al médico que no estoy conforme con los resultados del tratamiento (Seleccione una opción)

☐ Totalmente de acuerdo

☐ De acuerdo

☐ Ni de acuerdo ni en desacuerdo

☐ En desacuerdo

☐ Totalmente en desacuerdo

19. Evito hablar de cambios en el tratamiento por desconfianza o incomodidad (Seleccione una opción)

☐ Totalmente de acuerdo

☐ De acuerdo

☐ Ni de acuerdo ni en desacuerdo

☐ En desacuerdo

☐ Totalmente en desacuerdo

Sección V: Relación médico-paciente

20. Siento que mi médico escucha mis opiniones durante la consulta (Seleccione una opción)

☐ Totalmente de acuerdo

☐ De acuerdo

☐ Ni de acuerdo ni en desacuerdo

☐ En desacuerdo

☐ Totalmente en desacuerdo

21. Puedo hablar abiertamente con mi médico sobre cómo me siento con el tratamiento (Seleccione una opción)

☐ Totalmente de acuerdo

☐ De acuerdo

☐ Ni de acuerdo ni en desacuerdo

☐ En desacuerdo

☐ Totalmente en desacuerdo

22. Confío en que el profesional de salud me sugiere lo mejor para mi condición (Seleccione una opción)

☐ Totalmente de acuerdo

☐ De acuerdo

☐ Ni de acuerdo ni en desacuerdo

☐ En desacuerdo

☐ Totalmente en desacuerdo

23. Durante la atención médica me siento parte de las decisiones sobre mi tratamiento (Seleccione una opción)

☐ Totalmente de acuerdo

☐ De acuerdo

☐ Ni de acuerdo ni en desacuerdo

☐ En desacuerdo

☐ Totalmente en desacuerdo

Sección VI: Percepción del Sistema de salud

24. El sistema de salud retrasa la atención médica, lo que impide que mi tratamiento sea revisado a tiempo (Seleccione una opción)

☐ Totalmente de acuerdo

☐ De acuerdo

☐ Ni de acuerdo ni en desacuerdo

☐ En desacuerdo

☐ Totalmente en desacuerdo

25. He tenido dificultades para acceder a medicamentos cuando el médico ha indicado un cambio (Seleccione una opción)

☐ Totalmente de acuerdo

☐ De acuerdo

☐ Ni de acuerdo ni en desacuerdo

☐ En desacuerdo

☐ Totalmente en desacuerdo

26. Los tiempos de espera para consultas con especialistas afectan negativamente el seguimiento de mi enfermedad (Seleccione una opción)

☐ Totalmente de acuerdo

☐ De acuerdo

☐ Ni de acuerdo ni en desacuerdo

☐ En desacuerdo

☐ Totalmente en desacuerdo

27. Los trámites administrativos dificultan que reciba el tratamiento adecuado en el momento oportuno (Seleccione una opción)

☐ Totalmente de acuerdo

☐ De acuerdo

☐ Ni de acuerdo ni en desacuerdo

☐ En desacuerdo

☐ Totalmente en desacuerdo
